# Motor Behavior Selectively Inhibits Hair Cells Activated by Forward Motion in the Lateral Line of Zebrafish

**DOI:** 10.1016/j.cub.2019.11.020

**Published:** 2020-01-06

**Authors:** Paul Pichler, Leon Lagnado

**Affiliations:** 1 Sussex Neuroscience, School of Life Sciences, University of Sussex, Brighton BN1 9QG, UK

**Keywords:** zebrafish, lateral line, hair cell, efference copy, corollary discharge

## Abstract

How do sensory systems disambiguate events in the external world from signals generated by the animal’s own motor actions? One strategy is to use an “efference copy” of the motor command to inhibit the sensory input caused by active behavior [1]. But does inhibition of self-generated inputs also block transmission of external stimuli? We investigated this question in the lateral line, a sensory system that allows fish and amphibians to detect water currents and that contributes to behaviors such as rheotaxis [2] and predator avoidance [3, 4]. This mechanical sense begins in hair cells grouped into neuromasts dotted along the animal’s body [5]. Each neuromast contains two populations of hair cells, activated by deflection in either the anterior or posterior direction [6], as well as efferent fibers that are active during motor behavior to suppress afferents projecting to the brain [7, 8, 9, 10, 11, 12]. To test how far the efference copy signal modulates responses to external stimuli, we imaged neural and synaptic activity in larval zebrafish during fictive swimming. We find that efferents transmit a precise copy of the motor signal and a single spike in the motor nerve can be associated with ∼50% inhibition of glutamate release. The efference copy signal acted with high selectivity on hair cells polarized to be activated by posterior deflections, as would occur during forward motion. During swimming, therefore, “push-pull” encoding of stimulus direction by afferents of opposite polarity is disrupted while still allowing a subset of hair cells to detect stimuli originating in the external world.

## Highlights

• Efferents in neuromasts of the lateral line convey a close copy of the motor signal
• Efferents suppress hair cell glutamate release within 50 ms of first motor spike
• Efferent modulation is biased toward hair cells activated during forward motion
• Motor activity blocks “push-pull” signaling of stimulus direction

## Graphical Abstract

**Figure:**
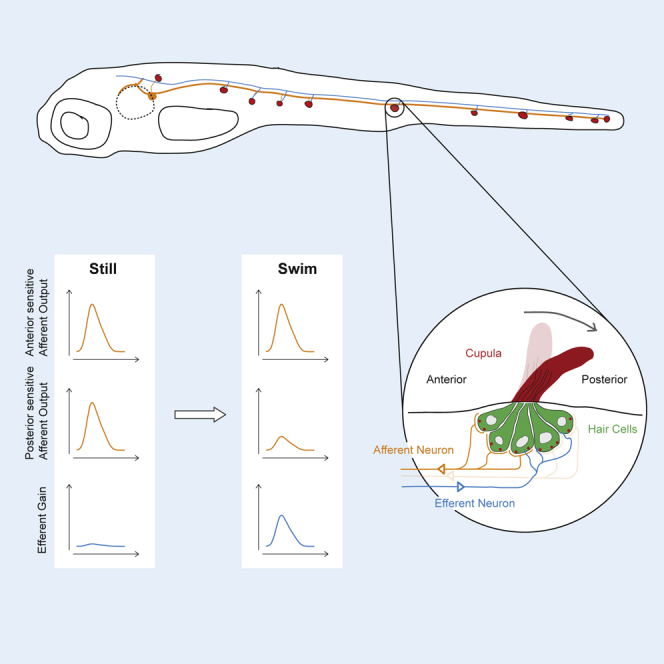

## Teaser

Pichler and Lagnado show that synaptic transmission from hair cells in the lateral line of zebrafish is suppressed by fictive swimming. Suppression is much stronger in hair cells activated during forward motion, so that “push-pull” signaling of stimulus direction is blocked while external stimuli can still be detected.

## Results

### Neuromasts Receive an Almost Exact Copy of the Motor Signal

**Figure 1:**
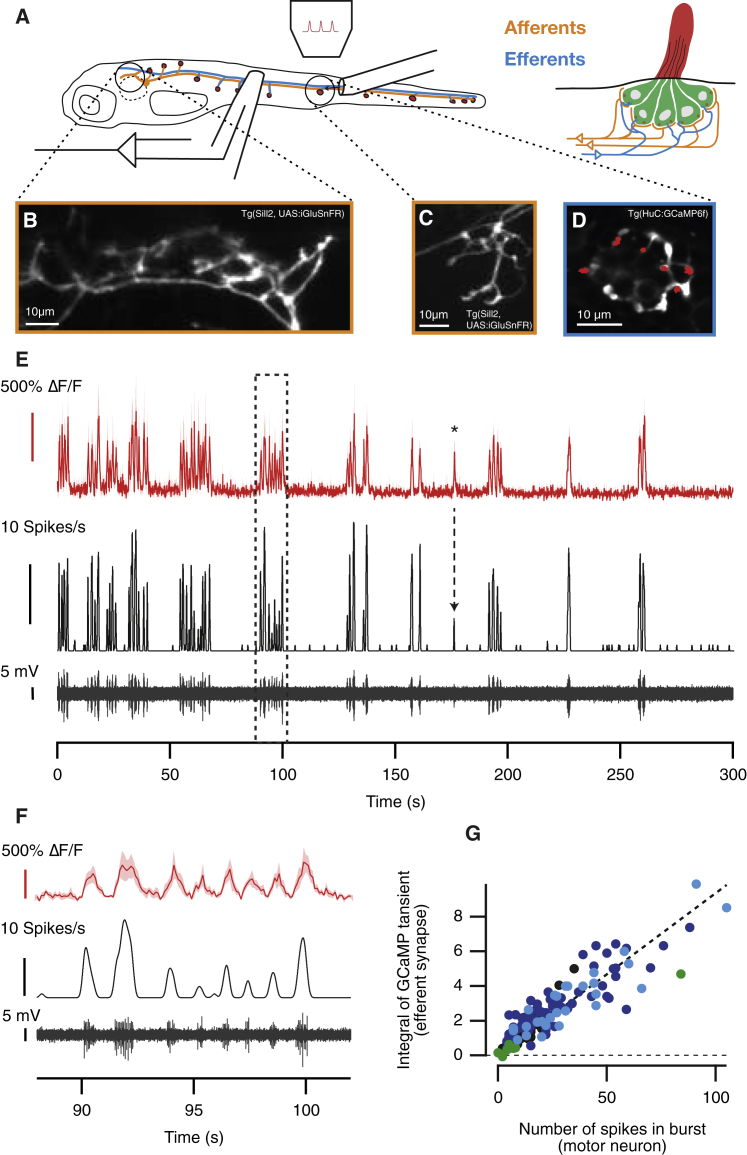
The Efferent Signal Is an Almost Exact Copy of the Motor Signal during Fictive Swimming (A) At 7 dpf, the posterior lateral line of larval zebrafish consists of 14 neuromasts on each side (red dots). Each neuromast is innervated by at least two afferent neurons (yellow) and a single cholinergic efferent (blue). The hair bundles of all hair cells are confined by a gelatinous structure called cupula (red in right panel). We imaged glutamate release of individual hair cells in a neuromast while measuring motor neuron activity through a suction pipette. A second pipette applied pressure steps to the neuromast. (B and C) Average projections of the afferent synapses in the hindbrain (B) and a neuromast (C) of a larva expressing iGluSnFR under transcriptional control of the Sill promoter (*Tg(Sill2, UAS:iGluSnFR)*). (D) Average projection of a neuromast in a larva expressing GCaMP6f under the transcriptional control of the HuC (elavl3) promoter (*Tg(HuC:GCaMP6f)*), in which afferents and efferents (but not hair cells) are labeled. Red dots indicate efferent regions of interest (ROIs) identified based on their firing pattern. (E) Top trace (red): “spontaneous” calcium transients in efferent synapses observed in the absence of mechanical stimulation (from D) over a 5-min period. The lower traces (black) depict the raw motor activity and the spike rate. The asterisk indicates a signal in the efferent synapses that correlates to six spikes in the motor nerve. (F) Magnified view of the dashed area in (E), showing that efferent synapses in the neuromast are activated at each swim bout. (G) The number of spikes per swimming bout and the integral of the fluorescent signal during that episode were strongly correlated (r = 0.9; n = 155 bouts from 4 neuromasts, each depicted in a different color). See also Figures S1 and S2.

Cholinergic efferents entering neuromasts are thought to be co-activated with motor neurons to provide feedforward control of the sensitivity of the lateral line [11, 13], but the quantitative relationship between motor activity and efferent and afferent signals are not known. To understand these aspects of the systems operation, we used an *in vivo* preparation of transgenic zebrafish larvae (5–9 days post-fertilization [dpf]) that undergo fictive swimming while neuromuscular transmission is blocked [14]. Motor nerve activity was measured electrophysiologically while optical reporters were used to monitor calcium signals in efferent and afferent neurons in neuromasts toward the back of the tail (the posterior lateral line). We also imaged glutamate release from ribbon synapses of hair cells and combined these various measurements with the application of mechanical stimuli to assess changes in sensitivity of the same neuromast to stimuli of different directions (Figures 1A–1D) [15].

To monitor the efferent signal, we used the *Tg(elavl3:GCaMP6f)* line of fish that express the calcium indicator GCaMP6f in afferent and efferent fibers, but not hair cells [16] (Figure 1D). Presynaptic boutons of efferent fibers could be distinguished from the postsynaptic varicosities of afferent neurons both by their smaller and rounder shape [17] (Figure 1D) and by the effects of a mechanical stimulus (afferents were excited although efferents were not affected; Figure S1). In all 15 neuromasts tested, fictive swimming caused efferent synapses to be activated in a burst-like fashion in close synchrony with spiking activity in the motor nerve (Figure 1E). These two signals were tightly coupled: each burst of spikes in the motor nerve was associated with a calcium transient in efferent synapses (Figure 1F), and the number of spikes in a bout was directly proportional to the time integral of the calcium signal (Figure 1G). As few as 6 spikes within a motor burst were correlated with a sizeable calcium signal in the efferent synapses (asterisk in Figure 1E). These results demonstrate that the efferent signal transmitted to the neuromast copies the motor signal driving locomotion both quantitatively and temporally. Notably, activity across all the efferent synapses within a single field of view were closely synchronized irrespective of the polarity of the hair cell contacted (Figure S2).

### The Efference Copy Suppresses Both Spontaneous and Stimulus-Evoked Transmission from Hair Cells

**Figure 2:**
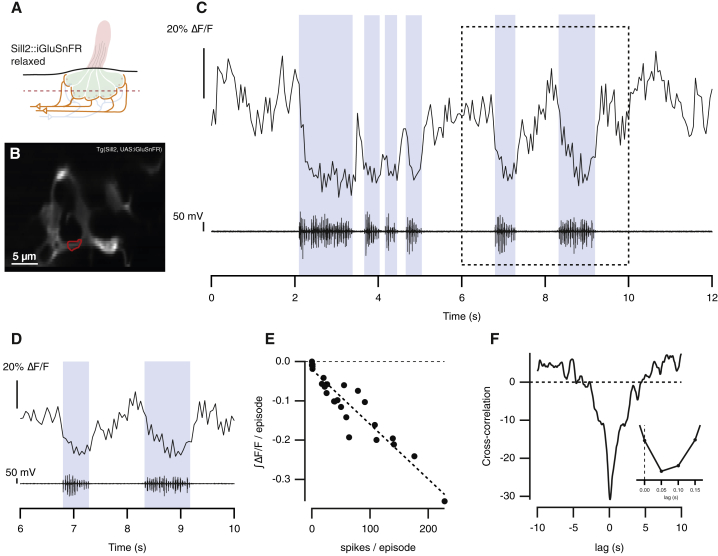
Spontaneous Release of Glutamate from Hair Cells Is Suppressed during Fictive Swimming (A) Experiments were carried out in “relaxed” mutants that express the glutamate reporter iGluSnFR in afferent neurons (*Tg(Sill2, UAS:iGluSnFR), cacnb*^*ts25/ts25*^) at 5 dpf. The dotted line represents the plane of imaging. (B) A representative hair cell synapse outlined in red. (C) Spontaneous glutamate release from the synapse in (B) (top) and motor neuron activity measured simultaneously (bottom). Blue areas indicate bursts of fictive swimming. (D) Magnified view of boxed area in (C). The maximum suppression of glutamate release was similar for each burst of motor activity. (E) Relationship between the number of spikes in a burst and the negative integral of the iGluSnFR signal from the neuromast depicted in (B) (n = 28 swimming episodes; r = −0.95). (F) Cross-correlation of iGlusnFR signal and the spike train in the motor nerve (down sampled to match imaging frequency). The inset shows that the maximum degree of anti-correlation occurred at a delay of 50 ms, indicating that the iGluSnFR signal fell within one frame interval of a spike in the motor nerve. See also Figure S3.

To what extent does the efference copy signal modulate the output from a neuromast? To investigate this question, we monitored the synaptic output from hair cells by expressing the glutamate sensor iGluSnFR [18] under the control of the *Sill* promoter [15, 19] (Figures 1B, 1C, 2A, and 2B). In these experiments, we did not paralyze fish by the usual method of applying the neuromuscular blocker α-BTX, because this agent has also been reported to block the α9/α10 isoforms of nicotinic acetylcholine receptors (nAChR) present in hair cells [20, 21]. Instead, we expressed iGluSnFR in the background of the *relaxed* mutant (cacnb^ts25/ts25^), in which defective dihydropyridine receptors block excitation-contraction coupling in muscles [22, 23, 24].

Bouts of fictive swimming reduced the spontaneous release of glutamate from hair cells occurring in the absence of a stimulus, as shown by the example in Figure 2C. Suppression was evident for each burst of motor activity (Figure 2D), and the integral of the decrease in the iGluSnFR signal during a burst was directly proportional to the number of spikes it contained (Figure 2E). Cross-correlating the iGluSnFR signal with the motor nerve recording revealed that suppression of synaptic transmission was maximal within 50 ms of a motor spike, which was the temporal resolution of image acquisition (Figure 2F). Substantial recovery of glutamate release from the hair cell occurred within 100 ms of the end of a burst of spikes (Figure 2D), demonstrating that inhibition was reversible on short timescales. Similar suppression of spontaneous activity was observed in four neuromasts out of eight, in which motor nerve activity occurred in the absence of mechanical stimulation. As expected, motor activity also suppressed synaptic transmission of spontaneous signals at the output of afferent neurons terminating in the medial octavolateralis nucleus (MON) (Figure S3). When an afferent signaling one direction of motion is activated, spontaneous activity in the afferent of opposite polarity is suppressed [5, 15]. Blocking spontaneous release will therefore disrupt “push-pull” signaling of stimulus direction during motor activity.

**Figure 3:**
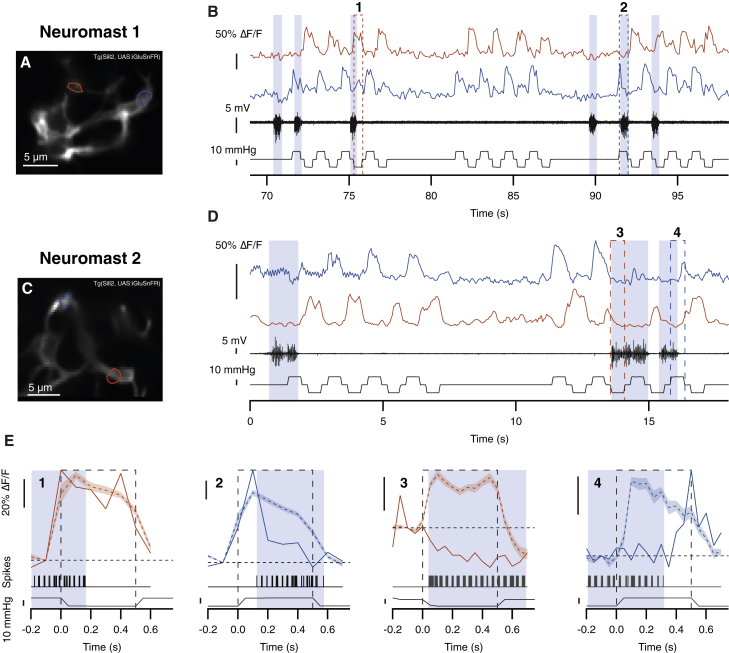
Motor Behavior Blocks Synaptic Transmission from a Subset of Hair Cells Experiments were carried out in relaxed mutants that express the glutamate reporter iGluSnFR in afferent neurons (*Tg(Sill2, UAS:iGluSnFR), cacnb*^*ts25/ts25*^) at 5 dpf. (A and B) Image of iGluSnFR expression in afferents of neuromast 1 (A). Two representative synaptic inputs are highlighted in red (activated by anterior deflection) and blue (activated by posterior deflection). The responses of these synapses to mechanical stimuli are shown in (B), together with motor nerve activity (black traces) and pressure steps applied to the neuromast. Positive pressure steps correspond to posterior deflections of the cupula and negative steps to anterior deflections. Blue shading indicates periods of motor nerve activity, and numbered boxes indicate the stimulation episodes that are magnified in (E). (C and D) A corresponding representation of hair cell activity in neuromast 2. (E) Expansion of records in boxes 1–4 in (B) and (D). The superimposed dashed red and blue traces indicate the average mechanically induced response of that synapse in the absence of motor nerve activity. Shaded areas represent the SEM. In example 2, inhibition of glutamate release is almost complete within 50 ms of the beginning of the motor burst. In example 3, suppression is complete within 50 ms, and further motor activity reduces glutamate release below resting levels. In example 4, glutamate release begins to recover within 50 ms of the end of the motor burst. See also Figure S4.

Stimulus-evoked release of glutamate from hair cells was also suppressed during fictive swimming. In these experiments, we stimulated individual neuromasts with positive and negative pressure steps that deflected the cupula along the anterior-posterior axis, thereby differentially activating the two populations of hair cells [15]. Simultaneous with stimulation, we measured motor nerve activity and synaptic release of glutamate onto afferent neurons (Figure 3). These pressure steps were sufficient to generate maximal responses, and examples of glutamate signals from two neuromasts are shown in Figures 3A–3D. For each neuromast, we show signals from two hair cells: one polarized to be excited by posterior deflections of the cupula (blue traces) and the other to anterior deflections (red traces). In neuromast 1, the hair cell signaling posterior deflections was markedly suppressed whenever mechanical stimulation overlapped with periods of motor nerve activity (Figure 3B, highlighted in blue). In contrast, the hair cell signaling anterior deflections was unaffected. Responses in the boxed areas “1” and “2” are shown on an expanded timescale in Figure 3E (left), where they have been superimposed on the average response of the same synapse in the absence of fictive swimming (dashed lines). In the affected hair cell, the iGluSnFR signal was strongly reduced within 50 ms of the onset of motor activity.

**Figure 4:**
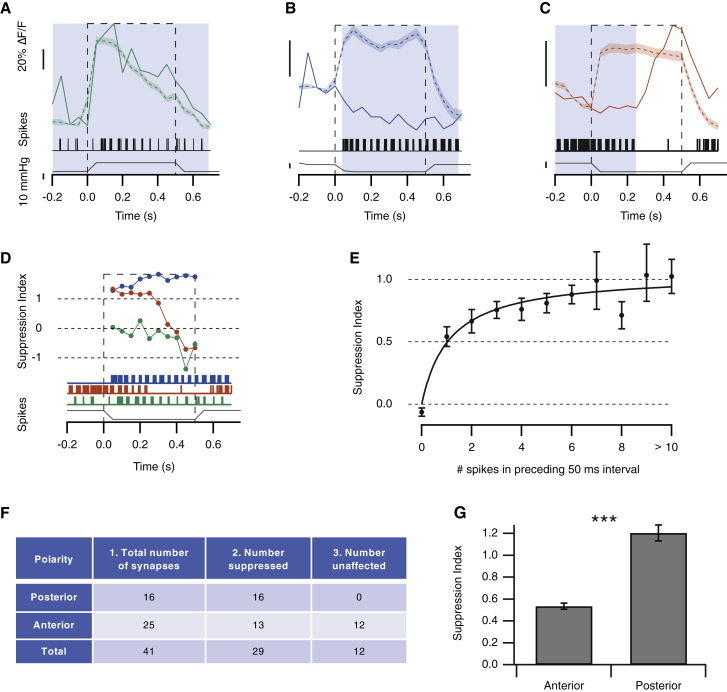
Motor Behavior Selectively Modulates Hair Cells Activated by Deflection in the Posterior Direction (A–C) Three examples of synapses whose response was (A) unaffected, (B) suppressed during the entire stimulation episode, and (C) suppressed only during the initial part of the stimulus (shaded areas represent the SEM). (D) The suppression index (SI), calculated on a point-by-point basis during mechanical stimulation (Equation 1). The red, blue, and green traces show synapses from three different hair cells, with the corresponding motor activity shown below. Glutamate release from the green synapse (A) was not significantly suppressed, with an SI ∼0 (negative values occur whenever the response during motor activity is larger than the average response in the absence of motor activity). Stimulated release from the red synapse (C) was nulled during motor activity (SI ∼1) but then recovered at the end of the burst of spikes. Glutamate release from the blue synapse (B) was reduced to below resting levels (SI > 1). (E) Plot of the relation between the SI at each time point during a mechanical stimulus and the number of spikes in the motor nerve in the preceding 50-ms time interval. Only synapses classified as suppressed were analyzed. Collected results from 29 synapses in 6 fish are shown. The data could be described by a Hill equation of the form SI(N_s_) = (SI_max_^∗^N_s_)/(N_s_ + N_1/2_), where N_s_ is the number of spikes, SI_max_ is the maximum SI (1.05 ± 0.08), and N_1/2_ is the number of spikes coinciding with half-maximal suppression (1.12 ± 0.42). Error bars show SEM. (F) The effects of motor activity on synaptic transmission from hair cells of opposing polarity. Column 1: the number of synapses activated by deflection in the posterior and anterior directions is shown. Measurements were made in a total of 41 synapses in 8 neuromasts in 6 fish. Column 2: the number of synapses suppressed during motor activity, classified as described in the STAR Methods, is shown. Column 3: the number of synapses unaffected by motor activity is shown. (G) Comparison of the average magnitude of the suppression index during a swimming bout in hair cells polarized for anterior and posterior deflection. We also compared the maximal SI values during a swimming bout: these were also significantly different in hair cells of opposite polarity (p < 0.001; Mann-Whitney U test). Bars show SEM.

In neuromast 2, the two hair cells polarized to anterior and posterior deflections were *both* suppressed during fictive swimming (Figure 3D and boxed areas “3” and “4” in Figure 3E). A particularly profound reduction in gain is evident in the examples highlighted in box 3, where the response to the mechanical stimulus was not simply nulled: glutamate release fell *below* the pre-stimulus baseline, indicating that the efference copy signal was strong enough to also block a relatively high rate of spontaneous synaptic activity. Again, the suppressive effect of the efference copy signal could also be observed at later stages of signal transmission through the lateral line: glutamatergic output from afferent projections to the MON was strongly suppressed (Figure S4). We surveyed the effects of motor activity on the output of 41 hair cell synapses (8 neuromasts in 6 fish) and classified each as being suppressed or unaffected (see STAR Methods). The response to a strong mechanical stimulus was inhibited in 71% of synapses (29/41; Figure 4).

The relationship between motor activity and glutamate release was quantified using a metric, the suppression index (SI), that was calculated for each application of a mechanical stimulus. If R_o_(*t*) is the average iGluSnFR signal at time *t* in the absence of motor activity and R_m_(*t*) is the signal during a single stimulus trial, then SI at each time t during the trial was calculated as

(Equation 1) $$\text{SI}\left(\text{t}\right)\;=\;\frac{\text{Ro}\left(t\right)\;-\text{Rm}\left(t\right)}{Ro\left(t\right)}.$$

Thus, SI = 0 indicates no suppression, SI = 1 indicates full suppression, and SI > 1 indicates suppression below the pre-stimulus baseline (i.e., inhibition of glutamate release occurring at rest as well as complete nulling of the stimulus-evoked response). We had limited control over the timing of fictive swimming relative to the application of the mechanical stimulus, so SI could only be calculated when the two overlapped. Three examples of the calculation of SI are shown in Figure 4D, based on responses shown in Figures 4A–4C. Although the example in green was unaffected, the example in blue was suppressed strongly to below baseline levels (SI > 1). In the example in red, the SI fell from ∼1 to zero within 50–100 ms of the end of motor activity, demonstrating that efferent modulation was rapidly reversible.

To assess how activity in the motor nerve was related to changes in the sensitivity of hair cells, we measured the SI at each time point during application of a mechanical stimulus and related this to the number of motor spikes in the preceding 50-ms time window. Figure 4E shows collected results from 29 synapses in 6 fish. Half-maximal suppression was associated with an average of just 1.1 spikes in the preceding 50-ms period, and a burst of 5 spikes was associated with an average of 80% suppression. Together, the results in Figures 1, 2, 3, and 4 demonstrate that motor activity acts rapidly and efficiently to block transmission of self-generated stimuli at the first synapse in the lateral line system.

### Efferent Modulation Is Strongly Biased toward Hair Cells Activated by Posterior Deflection

Ultrastructural studies indicate that *all* hair cells within a neuromast are innervated by efferent neurons [12], but we found that motor activity only inhibited transmission from ∼70% (29/41; Figure 4F). To investigate this apparent discrepancy, we asked whether the effect of motor activity might depend on the polarity of the hair cell and found that it did: whereas 16/16 synapses polarized to posterior deflection were suppressed during motor activity, only 13/25 polarized to anterior deflection were affected (Figure 4F). Considering all synapses irrespective of polarity, the average probability of suppression was 29/41 = 0.7. Taking the null hypothesis as polarity having no bearing on suppression, the probability of observing suppression in all 16 synapses would be expected to be p = 0.7^16^ = 0.003: the null hypothesis can therefore be rejected. Further, of the 29 hair cells in which motor activity exerted a significant suppressive effect, the SI was greater in synapses activated by posterior deflection of the cupula (SI = 1.20 ± 0.03) compared to those activated by anterior deflection (Figure 4G; SI = 0.54 ± 0.07; a difference significant at p < 0.0001 using Mann-Whitney U test). In other words, a burst of motor activity completely and selectively blocked transmission of a mechanical stimulus by the hair cells sensitive to posterior deflection of the cupula although those of opposite polarity were still capable of signaling a stimulus.

## Discussion

Modulation of sensory processing during motor behavior will necessarily involve a variety of mechanisms, depending on the sensory modality [13, 25, 26, 27, 28, 29, 30]. Using the lateral line of zebrafish, we find that efferents projecting to neuromasts are activated in tight synchrony with motor commands (Figure 1) to rapidly and reversibly suppress transmission of signals from hair cells (Figures 2 and 3) and afferents projecting to the central nucleus, the MON (Figures S3 and S4). Modulation was highly selective for hair cells activated by deflection of the cupula toward the tail (Figure 4), which was unexpected because efferent fibers appear to contact all hair cells irrespective of polarity [12, 16]. The observation that the population of hair cells activated by anterior deflection retains sensitivity to external stimuli is consistent with behavioral experiments showing that the lateral line allows the detection of predators during swimming [3, 31, 32]. It appears that efferent connections to hair cells are not equally effective in inhibiting synaptic transmission, possibly reflecting presynaptic differences in the efficiency with which spikes trigger acetylcholine release and/or postsynaptic differences in the density of nicotinic receptors mediating calcium influx or calcium-activated potassium channels causing hyperpolarization [33].

The lateral line contributes to reflexes such as rheotaxis, when the fish generates a forward swimming motion to stabilize itself against the water stream [2, 34]. Indeed, gentle swimming can be triggered by mechanical stimulation of a single neuromast [35]. These swimming reflexes will themselves result in stimulation of neuromasts, which would cause positive feedback if the efference copy signal did not break the loop by blocking transmission from hair cells activated by forward motion. Inhibiting the lateral line system at source—the hair cells—provides a mechanism that breaks this feedback loop while minimizing the activation of neurons downstream. A second advantage of efferent modulation may be to counteract adaptation caused by self-generated stimuli. During activity, depletion of vesicles at the ribbon synapse reduces the gain of transmission from hair cells [15, 36, 37, 38], and seconds of rest are required to recover the maximum gain of transmission after activity has stopped [39]. Blocking synaptic transmission from a subset of hair cells during motor activity will therefore also counteract the delayed effect of reduced sensitivity to external stimuli after a swimming bout.

The swimming motion of a larval zebrafish consists of a propulsion phase during which sharp undulatory movements of the tail accelerate the animal in a forward direction, and a glide phase, during which momentum carries the fish forward while its body is straight [30, 40]. Synaptic transmission from hair cells recovered ∼100 ms after the end of a burst of motor activity (Figures 3 and 4), indicating that the glide phase will be subject to significantly less efferent inhibition than the propulsion phase. The patterns of water flow along the surface of the tail will be complex during undulatory tail motions [41, 42], and it seems likely that hair cells polarized to be activated by anterior deflection of the cupula will be stimulated even when those polarized for posterior deflections are inhibited. The pattern of activity emerging from the neuromast during motor activity will, however, differ from that generated by an external stimulus in a fundamental way: afferents activated by anterior and posterior deflection will no longer vary in antiphase and the usual “push-pull” signaling of direction will be lost.

The comparison of signals through neurons of different tuning, often by a mechanism of lateral inhibition, is a common strategy by which sensory systems discriminate between different stimuli. Central mechanisms might therefore also contribute to distinguishing between self-generated stimuli and those originating in the external world. The MON, for instance, where afferent neurons from the anterior as well as the posterior lateral line terminate, is a “cerebellum-like” structure thought to be involved in the adaptive filtering of self-generated sensory information [43, 44]. It will be interesting to investigate how the central mechanisms generating motor reflexes are altered by disrupting the representation of stimulus direction within the lateral line.

## STAR★Methods

### Key Resources Table

**Table:** 

REAGENT or RESOURCE	SOURCE	IDENTIFIER
**Chemicals, Peptides, and Recombinant Proteins**		

α-Bungarotoxin	Tocris Bioscience	Cat. No. 2133
MS-222 (Tricaine methanesulfonate)	Merck	E10521

**Experimental Models: Organisms/Strains**		

Tg(elavl3:GCaMP6f)	Lab of Isaac Bianco	ZFIN ID: ZDB-FISH-160927-3
Tg(Sill2, UAS:iGluSnFR)	Lab of Leon Lagnado	n/a
*Tg(Sill2, UAS:iGluSnFR), cacnb*^*ts25/ts25*^	Lab of Leon Lagnado	n/a

### Lead Contact and Materials Availability

Further information and requests for resources and reagents should be directed to and will be fulfilled by the Lead Contact, Leon Lagnado (l.lagnado@sussex.ac.uk).

### Experimental Model and Subject Details

#### Zebrafish

All procedures were in accordance with the UK Animal Act 1986 and were approved by the Home Office and the University of Sussex Ethical Review Committee.

Three zebrafish lines were used in this study. (1) The *Tg(HuC:GCaMP6f)* expresses GCaMP6f in all neurons (except for a small number of neuronal sub-types, including hair cells) and was kindly provided by Dr Isaac Bianco. (2) The *Tg(Sill2, UAS:iGluSnFR)* expresses the glutamate sensor iGluSnFR [18] under the control of the Sill promoter, which specifically targets afferent neurons of the posterior and anterior lateral line [19] and allows to measure hair cell glutamate release onto the afferents in the neuromast, as well as glutamate release by the afferent neurons in the hindbrain [15]. (3) The (*Tg(Sill2, UAS:iGluSnFR), cacnb*^*ts25/ts25*^*)*, is the same as (2) only in the background of the ‘*relaxed’* (cacnb^ts25/ts25^) mutation [22, 23], which yields immotile homozygotes, due to a point-mutation in the b1a subunit of the dihydropyridine receptor involved in excitation-contraction coupling of skeletal muscle. It was generated by co-injecting the Sill2 and the 10xUAS:iGluSnFR plasmids (12 ng/μl) as well as the Tol2 transposase (40 ng/μl) [45] into one-cell stage embryos originating from an in-cross of heterozygous *relaxed* mutants (cacnb^ts25/+^). Larvae were screened for expression of the iGluSnFR transgene and reared to adulthood. Founder fish, heterozygous for the *relaxed* mutation and carrying the Sill2 and iGluSnFR transgenes in their germline, were identified by outcrossing to heterozygous *relaxed* fish and screening the offspring for immobility (only the homozygotes are immotile) as well as the expression of iGluSnFR in lateral line afferents. As the homozygous *relaxed* larvae are not viable and die at 5 - 6 days post fertilization (dpf), the line was maintained in a heterozygous background and in-crossed to yield homozygotes, necessary for experiments.

Adult zebrafish were maintained in fish water at 28.5°C under a 14:10 hour light:dark cycle under standard conditions [46]. Fish were bred naturally, and fertilized eggs were collected, washed with distilled water and transferred into 50 mL of E2 medium (concentrations in mM: 0.05 Na2HPO4, 1 MgSO4 7H2O, 0.15 KH2PO4, 0.5 KCl, 15 NaCl, 1 CaCl2, 0.7 NaHCO3, pH7-7.5). At 24 hours post fertilization (hpf) 1-phenyl2-thiourea (pTU) was added to yield a final concentration of 0.2 mM to inhibit pigment formation.

### Method Details

#### Sample preparation

Sample preparation differed slightly between larvae in the wild-type background (*Tg(HuC:GCaMP6f), Tg(Sill2, UAS:iGluSnFR)*) and those in the immotile *relaxed* background (*Tg(Sill2, UAS:iGluSnFR), cacnb*^*ts25/ts25*^). Larvae of undetermined sex were used in all experiments. Experiments were performed between 7-9 dpf, on larvae that were screened for the strongest expression of the respective transgene. They were anaesthetized in in 0.016% tricaine (MS-222) and were placed ‘side-down’ in a ‘fish-shaped’ pit, carved out of a thin layer of PDMS (Sylgard184, Dow Crowning) on a coverslip and held down by a ‘harp’ (Warner Instruments). Pressure of the Nylon strings was adjusted so that blood circulation was not compromised. Then, 0.25 mM α-Bungarotoxin (Tocris Bioscience) was injected into the heart to induce paralysis. Special care was taken to not touch the upward facing side of the fish, to avoid damaging the cupula.

The inhibitory effects of α-Bungarotoxin on α9 nicotinic acetylcholine receptor subunits have been shown to be reversible within 10 minutes in an oocyte expression system [47]. We could not, however, exclude the possibility that injecting α-Bungarotoxin into the heart of the live animal would have residual effect on the physiology of lateral line hair cell. We therefore also carried out imaging experiments in fish paralyzed without the tricaine or α-Bungarotoxin. These were homozygous larvae of the *relaxed* line with a mutation in dihydropyridine receptor 1 [48], which were imaged at 5 dpf.

#### Two-Photon Imaging

Two-photon imaging was performed using a custom built two-photon microscope driven by a mode-locked Titanium-sapphire laser (Chameleon 2, Coherent) tuned to 915 nm [49] was used. In experiments on larvae of the wild-type background excitation was delivered through a 40x water immersion objective (Olympus, 40x LUMIPlanF, NA: 0.8) and in experiments on the *relaxed* larvae, a 25 x objective (Nikon N25X-APO-MP 1.1NA) was used. To improve the signal-to-noise emitted photons were collected through the objective as well as through an oil condenser (NA 1.4, Olympus), below the sample. Green emission filters (525/70 nm at the objective and 530/60 nm at the condenser) were used in front of GaAsP photodetectors (H10770PA-40, Hamamatsu). The photocurrents of the two detectors were summed and passed through a transimpedance amplifier (Model SR570, Stanford Research Systems) and low-pass filtered (300 kHz). The microscope was controlled by ScanImage v3.8 [50], synchronized with the stimulus application and operated at acquisition rates of 20-50 Hz. In this study, only neuromasts from the posterior lateral line (L3 – L6) with a directional sensitivity along the anterior-posterior axis were examined.

#### Mechanical stimulation

Neuromasts were stimulated with positive and negative pressure steps, applied through a glass pipette (GC150T-10, Harvard Apparatus) with a tip diameter of ∼30 μm, attached to a high-speed pressure clamp (HSPC-1, ALA scientific) [15, 51]. Output pressure was controlled through mafPC (courtesy of M. A. Xu-Friedman) running on IgorPro (Wavemetrics), which also triggered acquisition in ScanImage via a TTL pulse. The pipette tip, which was bent through ∼30° using a micro forge (Narishige) to stimulate the neuromast approximately parallel to the body surface of the fish, was positioned ∼20 μm above the body and ∼100 μm away from the neuromast. The pressure clamp was manually zeroed before the start of an experiments so that no net flow was produced. We chose stimulus strengths that elicited near saturating responses in hair cells, assessed by a coarse protocol consisting of three positive and negative pressure steps of increasing amplitude [15].

#### Visual stimulation

We engaged the optomotor response by projecting a moving grating directly onto the larva, moving in the tail to head direction (12 mm wide bars at 100% contrast that moved at 5 mm/s). A microprojector (Pico PK320, Optoma) from which the blue and green LED channels were removed was used at an intensity that did not lead to bleed-through in the photo-multiplier tubes. The visual stimulus was controlled via the PsychoPy toolbox running in Python 3.6 and synchronized to mafPC, controlling the mechanical stimulus, via a TTL pulse. This stimulus was not as efficient in triggering fictive locomotion in the set of experiments using *relaxed* larvae because these involved use of a wider objective that partially restricted light from the projector that actually reached the larvae.

#### Motor nerve recordings

Motor nerve recordings were performed with only minor modifications from previous work [14]. Recording electrodes were pulled to a tip diameter of ∼30 μm (from borosilicate glass, GC150T-10, Harvard Apparatus) and subsequently fire polished using a micro forge (Narishige). It was filled with extracellular recording solution (concentrations in mM: 134 NaCl, 2.9 KCl, 1.2 MgCl2, 2.1 CaCl2, 10 HEPES buffer, adjusted to pH 7.8 with NaOH). The pipette was positioned dorsally of the larva, above myotomal cleft 8-14 at a 45° angle and perpendicular to the longitudinal body axis. Using a plastic syringe, slight positive pressure was applied during the approach and upon contacting the skin changed to negative pressures between −30 and −70 mmHg. On average, spontaneous motor nerve activity could be observed after 10-15 minutes. Using a BVC-700A (Dagan, USA) in current-clamp mode the extracellular voltage was measured. The signal was filtered (Brownlee model 440, Neurophase) with a high and low-pass cut off frequency of 300 Hz and 1 kHz, respectively and recorded using mafPC at a sample rate of 5 kHz (synchronously with the mechanical stimulation).

### Quantification and Statistical Analysis

#### Image segmentation and analysis

Images sequences (movies) were analyzed in Igor Pro. Small drifts in the x/y dimension were registered, using the SARFIA toolbox [52]. Movies with large drifts, and potential z-drifts were discarded. Regions of Interest (ROIs) were determined using an algorithm that identifies pixels with the highest correlation value to neighboring pixels as ‘seeds’ and then extends these to form ROIs based on a threshold defined by the experimenter [53]. These ROIs corresponded to sites of maximal glutamate release which occur in apposition to hair cell ribbon synapses [15].

Background fluorescence was subtracted manually. Baseline fluorescence (F) was defined as the average fluorescence in the first 10 s of imaging and preceding the first stimulation interval; the ratio of change in fluorescence (ΔF) was calculated relative to that value (ΔF/F) and used for further analysis. Contrast in images was adjusted for presentation purposes. The motor nerve recordings were further digitally filtered (300 Hz high-pass 1kHz low-pass and 50 Hz notch). Spikes were extracted using a custom written procedure that applied a simple threshold to the filtered signal and detected when it was crossed by the signal. This temporal filter was a Gaussian with FWHM = 100 ms.

#### Classification of suppressed synapses

Each synapse was classified as either ‘unaffected’ or ‘suppressed by efferent activity’ in three steps. First, we extracted the iGluSnFR trace that coincided with mechanical stimulation, leaving out the first point because of uncertainties as to its precise timing within an imaging frame. Second, each point in the iGluSnFR trace was put into one of two populations: those that coincided with at least one spike in the motor nerve recording and those that did not. Finally, these two populations were compared using a one-tailed non-parametric Mann-Whitney U-test and a significance level α = 0.05. The Mann-Whitney U test supposes independent data points, which appears a reasonable approximation given that the suppressive effects of efferent activity reversed within 1-2 imaging frames (50 – 100 ms). This assumption was conservative given that weak correlations between successive points in the iGluSnFR would tend to bias toward the null-hypothesis that the two populations are not different.

### Data and Code Availability

The datasets and code supporting the current study have not been deposited in a public repository because extensive explanation would be required to make them useful but they are available from the corresponding author on request.

## Supplemental Information

Document S1. Figures S1–S4

Document S2. Article plus Supplemental Information
