# Supplementary material for: Motor Behavior Selectively Inhibits Hair Cells Activated by Forward Motion in the Lateral Line of Zebrafish
Source: Curr Biol. 2020 Jan 6;30(1):150–157.e3. doi: 10.1016/j.cub.2019.11.020 (PMC6947483; doi:10.1016/j.cub.2019.11.020)
Supplement: Document S1. Figures S1–S4 [file mmc1.pdf]

Current Biology, Volume *30*

Supplemental Information

Motor Behavior Selectively Inhibits Hair Cells Activated by Forward Motion in the Lateral Line of Zebrafish

Paul Pichler and Leon Lagnado

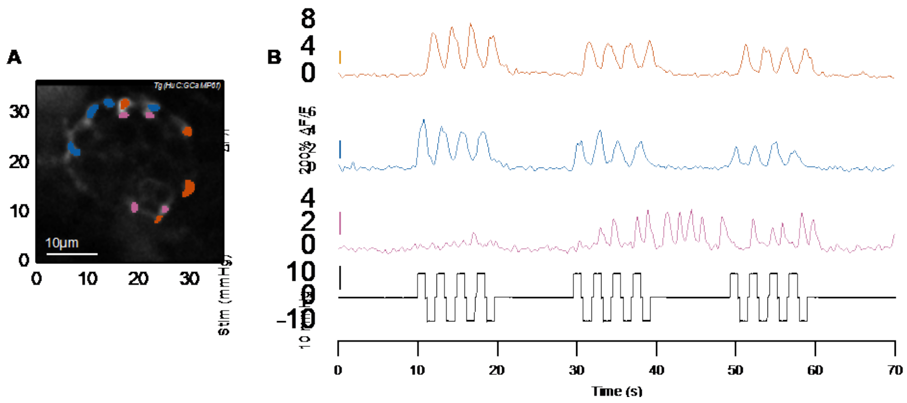

Figure S1. Efferent synapses can be identified based on their morphology and firing pattern, which is independent of mechanical stimulation. Related to Figure 1.

(A) Zebrafish larvae expressing the calcium indicator GCaMP6f under the control of the HuC promoter (*Tg(HuC:GCamP6f)*) were paralyzed with α-BTX. An average projection of a NM, highlighting irregularly shaped varicosities belonging to two afferents with opposing directional sensitivity (orange and blue) as well as small and round efferent boutons (magenta).

(B) The average signals from the respective ROIs in (A). Orange ROIs are sensitive to posterior deflections and blue ROIs to anterior ones, confirming that they are afferents. Magenta ROIs fired independent of the mechanical stimulation, confirming that they were efferent. Shaded area represents SEM.

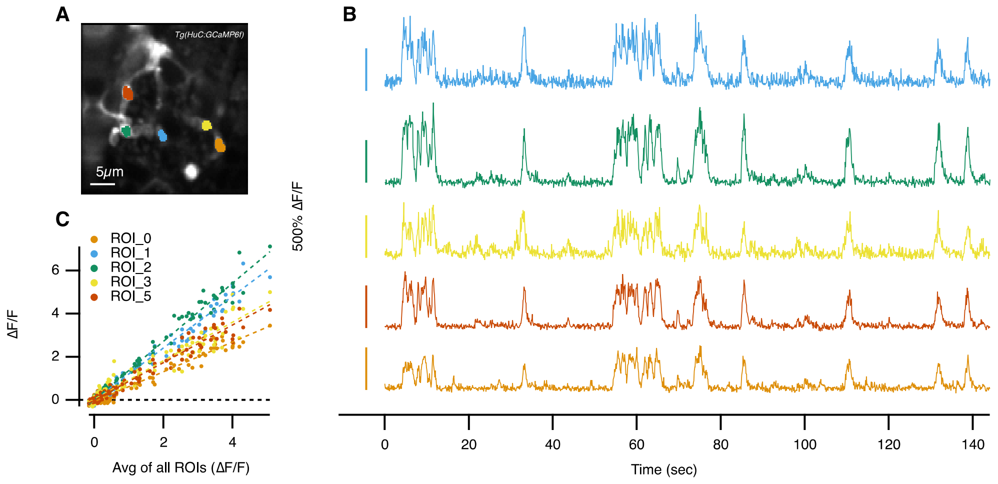

Figure S2. Activity is strongly synchronized across efferent synapses. Related to Figure 1.

(A) Zebrafish larvae expressing the calcium indicator GCaMP6f under the control of the HuC promoter (*Tg(HuC:GCamP6f)*) were paralyzed with α-BTX. Efferent ROIs were identified based on their, small, roundish morphology as well as their 'spontaneous' activity in the absence of mechanical stimulation.

(B) Response profile of the 5 ROIs depicted in (A) over a 145 s time window. These were all "spontaneously" active in the absence of mechanical stimulation.

(C) Plot of the instantaneous signal in each of the five synapses as a function of the average activity of all five. Activity in B was down-sampled into time bins of 2 s. Each set of data points could be fit by a straight line through the origin with r > 0.9 revealing a high degree of synchronicity.

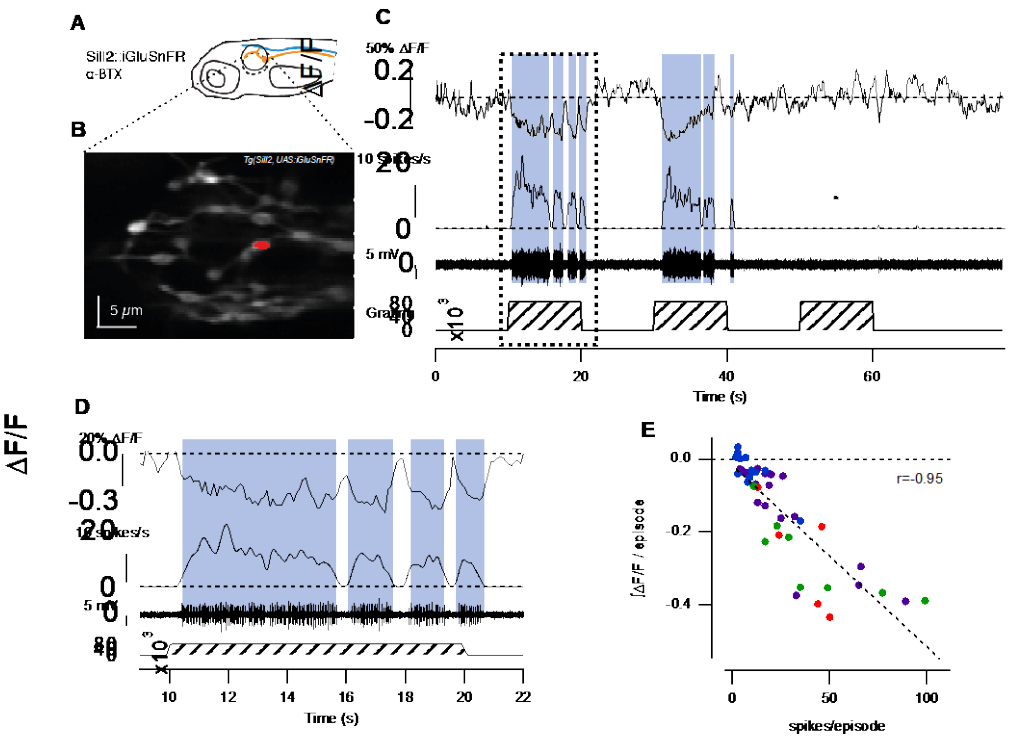

Figure S3. Motor activity suppressed spontaneous activity in afferent fiber synapses transmitting to the medial octavolateralis nucleus (MON). Related to Figure 2.

(A) Experiments were carried out in larvae that expressed the glutamate reporter iGluSnFR in afferent neurons (*Tg(Sill2, UAS:iGluSnFR*) and which were paralyzed with α-BTX.

(B) An average projection of the posterior arm of the MON, highlighting the synapse whose response is depicted in (C). (**C**) An example of a synapse, whose baseline glutamate release, in the absence of a mechanical stimulus, was suppressed by fictive swimming (blue-shaded area). The absence of suppression during the third presentation of a visual grating (*) indicated that the visual stimulus did not directly affect the encoding of mechanical information. (**D**) Magnification of the dashed box in (C) reveals that each individual swim- burst leads to a transient suppression of the glutamate release. (**E**) The number of spikes in the motor neuron during a swim-bout (episode) and the integral of the suppressive effect in the hindbrain synapse is tightly correlated (r=-0.95, n=49 bouts from 4 synapses).

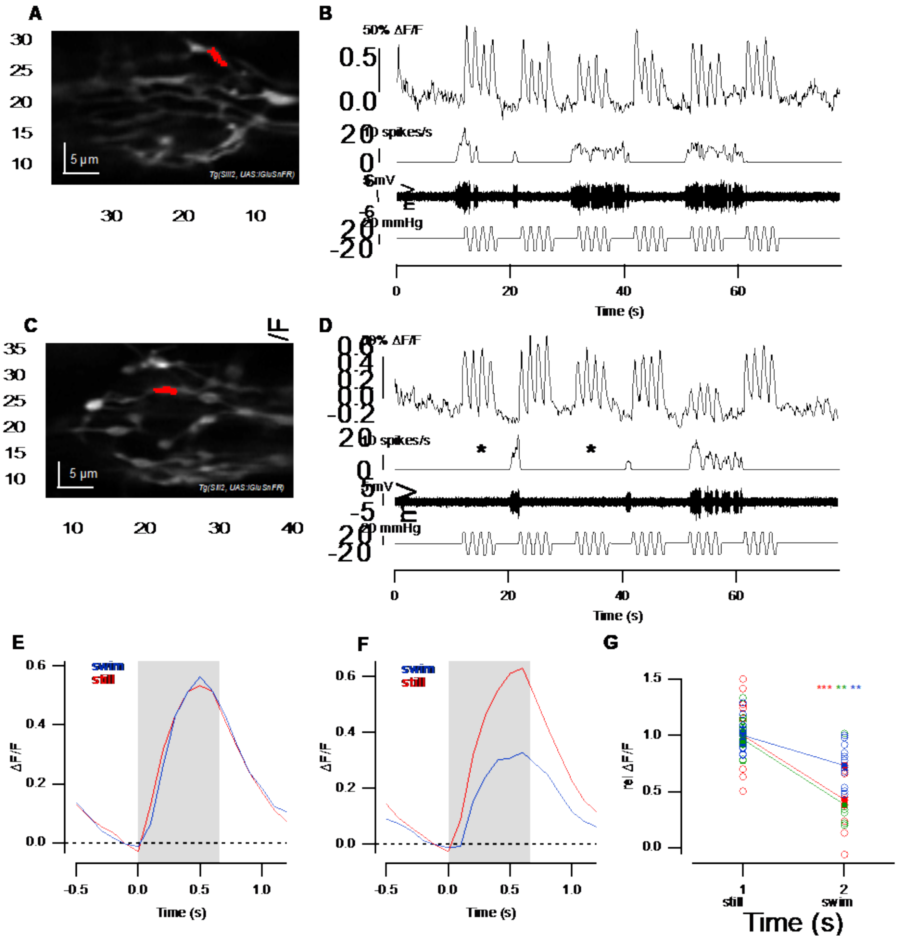

Figure S4. Motor activity suppressed stimulus-evoked activation of afferent fiber synapses transmitting to the medial octavolateralis nucleus (MON) in the hindbrain. Related to Figure 3.

Experiments were carried out in larvae that expressed the glutamate reporter iGluSnFR in afferent neurons (*Tg(Sill:Gal4, UAS:iGluSnFR*) and which were paralyzed with α-BTX. A given neuromast was stimulated with positive and negative pressure steps while the output of afferent neurons in the MON was imaged with a two-photon microscope. (**A** and **C**) Average projections of synapses in the posterior part of the MON. Highlighted in (A) is the synapse whose response is depicted in (B) and highlighted in (C) is the synapse whose response is depicted in (D). (**B** and **D**) Two representative examples of afferent synapses in the MON, which are sensitive to posterior deflections. The bottom trace represents the mechanical stimulation of the NM (positive pressure corresponds to posterior and negative pressure steps to anterior deflection of the cupula, respectively). The middle two traces depict the raw motor signal and average spike rate, respectively, and the top trace shows the synaptic activity of the synapse. Blue-shaded areas indicate periods in which mechanical

stimulation coincided with fictive swimming and red-shaded areas indicate periods in which it did not. The first third and fifth mechanical stimulation period always overlapped with the presentation of a moving grating to induce fictive locomotion. (**B**) The activity of this synapse was not affected by fictive locomotion. This is quantified in (E), which depicts the mean responses of this synapse in the presence and absence of fictive locomotion. (**D**) Example of a synapse whose response to mechanical stimulation was suppressed when it coincided with fictive locomotion, quantified in (F). Asterisks indicate periods in which visual stimulation failed to induce locomotion. (**F**) The peak amplitude of the IGluSnFR signal in the MON was reduced by 42% during fictive locomotion (P<0.005, Mann-Whitney U-test). (**G**) The mechanically induced iGluSnFR signal in three hindbrain synapses while still (left) and during 'fictive swimming' (right). All these afferents were activated by posterior deflections of the cupula. Open circles represent the response to individual stimulations and filled circles their average (*** P<0.0001, ** P<0.001, Mann-Whitney U-test). (Shaded areas in (E) and (F) represent the SEM).
